# Rapid Clinical Screening of Burkholderia pseudomallei Colonies by a Bacteriophage Tail Fiber-Based Latex Agglutination Assay

**DOI:** 10.1128/AEM.03019-20

**Published:** 2021-05-26

**Authors:** Veerachat Muangsombut, Patoo Withatanung, Narisara Chantratita, Sorujsiri Chareonsudjai, Jiali Lim, Edouard E. Galyov, Orawan Ottiwet, Sineenart Sengyee, Sujintana Janesomboon, Martin J. Loessner, Matthew Dunne, Sunee Korbsrisate

**Affiliations:** a Department of Immunology, Faculty of Medicine Siriraj Hospital, Mahidol University, Bangkok, Thailand; b Department of Microbiology and Immunology, Faculty of Tropical Medicine, Mahidol University, Bangkok, Thailand; c Department of Microbiology and Melioidosis Research Center, Faculty of Medicine, Khon Kaen University, Khon Kaen, Thailand; d DSO National Laboratories, Singapore; e Department of Genetics and Genome Biology, College of Life Sciences, University of Leicester, Leicester, United Kingdom; f Department of Medical Technology and Clinical Pathology, Mukdahan Hospital, Mukdahan, Thailand; g Institute of Food Nutrition and Health, ETH Zurich, Zurich, Switzerland; The Pennsylvania State University

**Keywords:** *Burkholderia pseudomallei*, bacteriophage, latex agglutination assay, melioidosis, rapid colony screening, tail fiber, tail assembly chaperone

## Abstract

Melioidosis is a life-threatening disease in humans caused by the Gram-negative bacterium Burkholderia pseudomallei. As severe septicemic melioidosis can lead to death within 24 to 48 h, a rapid diagnosis of melioidosis is critical for ensuring that an optimal antibiotic course is prescribed to patients. Here, we report the development and evaluation of a bacteriophage tail fiber-based latex agglutination assay for rapid detection of B. pseudomallei infection. *Burkholderia* phage E094 was isolated from rice paddy fields in northeast Thailand, and the whole genome was sequenced to identify its tail fiber (94TF). The 94TF complex was structurally characterized, which involved identification of a tail assembly protein that forms an essential component of the mature fiber. Recombinant 94TF was conjugated to latex beads and developed into an agglutination-based assay (94TF-LAA). 94TF-LAA was initially tested against a large library of *Burkholderia* and other bacterial strains before a field evaluation was performed during routine clinical testing. The sensitivity and specificity of the 94TF-LAA were assessed alongside standard biochemical analyses on 300 patient specimens collected from an area of melioidosis endemicity over 11 months. The 94TF-LAA took less than 5 min to produce positive agglutination, demonstrating 98% (95% confidence interval [CI] of 94.2% to 99.59%) sensitivity and 83% (95% CI of 75.64% to 88.35%) specificity compared to biochemical-based detection. Overall, we show how a *Burkholderia*-specific phage tail fiber can be exploited for rapid detection of B. pseudomallei. The 94TF-LAA has the potential for further development as a supplementary diagnostic to assist in clinical identification of this life-threatening pathogen.

**IMPORTANCE** Rapid diagnosis of melioidosis is essential for ensuring that optimal antibiotic courses are prescribed to patients and thus warrants the development of cost-effective and easy-to-use tests for implementation in underresourced areas such as northeastern Thailand and other tropical regions. Phage tail fibers are an interesting alternative to antibodies for use in various diagnostic assays for different pathogenic bacteria. As exposed appendages of phages, tail fibers are physically robust and easy to manufacture, with many tail fibers (such as 94TF investigated here) capable of targeting a given bacterial species with remarkable specificity. Here, we demonstrate the effectiveness of a latex agglutination assay using a *Burkholderia*-specific tail fiber 94TF against biochemical-based detection methods that are the standard diagnostic in many areas where melioidosis is endemic.

## INTRODUCTION

Burkholderia pseudomallei is an environmental saprophyte bacterium that causes melioidosis, a disease endemic to northern Australia, Southeast Asia, and other tropical regions (1), with northeastern Thailand reporting the highest rate of infections (2). Humans and animals acquire B. pseudomallei infections through wounds or inhalation or ingestion of contaminated soil or surface water (1). Every year there are an estimated 165,000 melioidosis cases, causing 89,000 deaths (54% mortality) worldwide (1). There are no vaccines or other approved prophylactics available to prevent B. pseudomallei infections (3). Due to the clinical severity and high case-fatality rate of melioidosis, a prompt and accurate identification of B. pseudomallei is of paramount importance to ensure optimal medical treatment.

Conventional B. pseudomallei identification involves bacterial isolation, cultivation, and characterization of clinical samples using biochemical analyses, which can take several days to provide results (4). In addition, nucleic acid-based detection, MALDI-TOF (matrix-assisted laser desorption/ionization time-of-flight), and antibody-based detection methods are revolutionizing pathogen identification (5–7); however, their wide-spread use within developing countries can sometimes be limited due to running costs and infrastructure requirements. Presently, several immunological techniques are available for B. pseudomallei detection, including latex agglutination assays (LAAs) (8–10) and lateral flow immunoassays (LFIs) such as the Active Melioidosis Detect (InBios International, USA) (11–13) that use antibodies targeting lipopolysaccharide (LPS) or capsular polysaccharide (CPS) antigens. The combination of LAAs with biochemical methods has proven highly effective for B. pseudomallei identification, especially within Thailand (8, 14). Unfortunately, LAAs commonly demonstrate cross-reactivity to nontarget bacteria, such as Staphylococcus aureus and the related B. cepacia complex (15, 16), leading to misdiagnosis and incorrect antibiotic treatment. Thus, development of diagnostic assays with antibody-alternative bio-probes have the potential to improve the sensitivity and specificity of LAAs and other antibody-based assays.

Bacteriophages (phages) are viruses that infect a limited range of bacteria (17) and have been previously investigated as therapeutic agents to treat *Burkholderia* infections (18–20). Phages mediate recognition of bacterial hosts via specialized receptor binding proteins (RBPs), i.e., tail fibers and tailspikes (21). The high binding specificity of RBPs makes them ideal bio-probes for bacterial diagnostics (22, 23). As exposed appendages, RBPs are extremely robust and typically feature high resistances to salts, proteases, and temperature or pH changes while remaining capable of specific binding in harsh environmental conditions.

Here, we report the isolation and characterization of *Burkholderia* phage E094 and the development of its tail fiber (94TF) into an LAA for rapid B. pseudomallei identification. The assay demonstrated high sensitivity and specificity compared to those of conventional biochemical analyses.

## RESULTS

### Isolation, characterization, and genome sequencing of *Burkholderia* phage E094.

**FIG 1:**
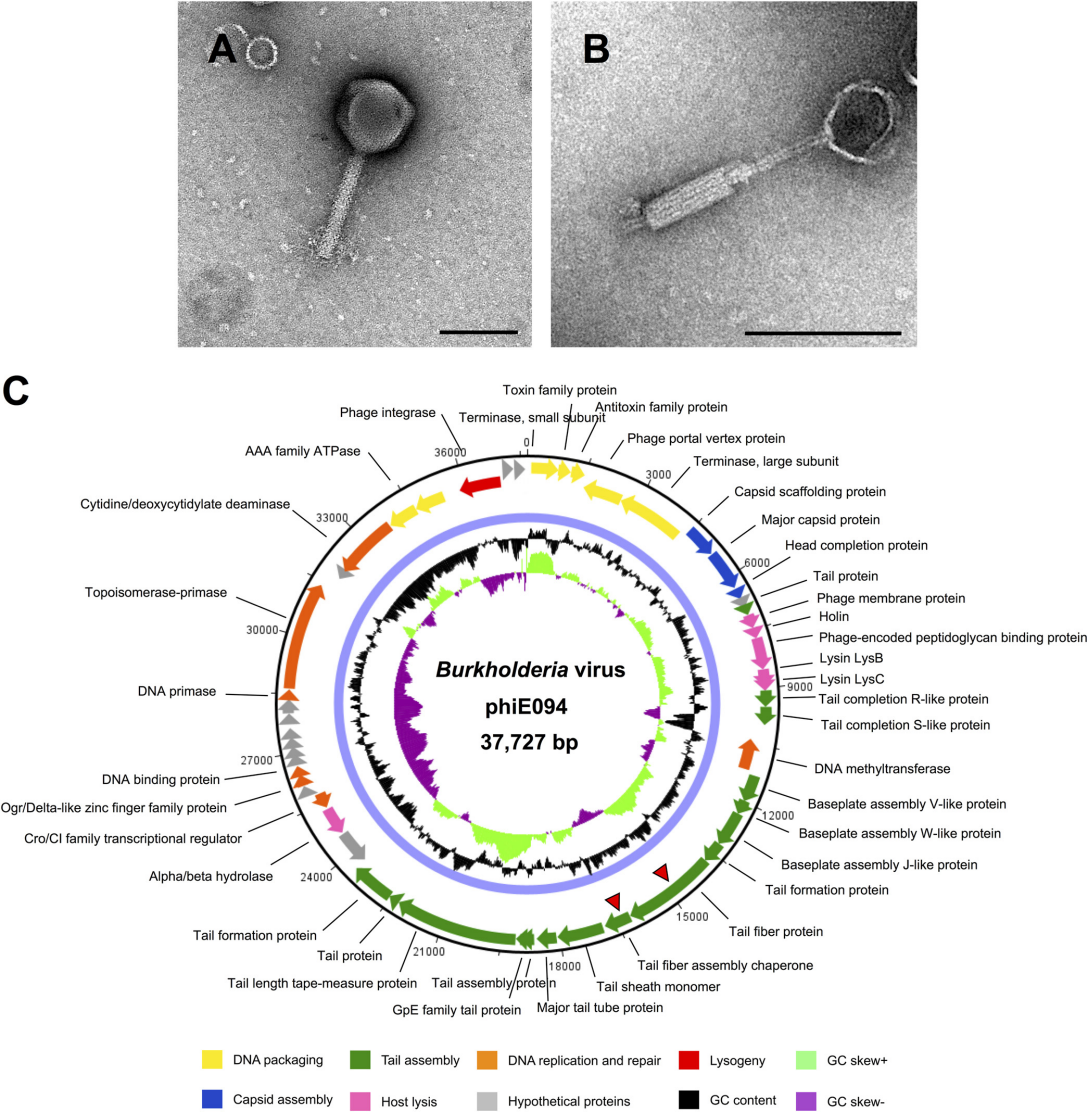
Morphology and genome of *Burkholderia* virus phiE094. TEM analysis of phage E094 with its tail in the noncontracted (A) and contracted (B) states confirmed its myoviral classification. Scale bar, 100 nm. (C) Genome of *Burkholderia* virus phiE094. The genome was generated using Artemis version 18.1.0 and is annotated and colored based on predicted molecular functions of identified genes. Gp23 and gp24 are identified by two red triangles. The center of the genome map provides % GC content (black) and the GC skew+ and skew− are shown in light green and purple, respectively.

Phage E094 was isolated from soil collected from Ubon Ratchathani province. As B. pseudomallei requires a biosafety level 3 laboratory, the nonpathogenic, surrogate species B. thailandensis DV1 was used for phage propagation (24). E094 infected all 14 B. thailandensis strains and 5/8 B. pseudomallei stains initially tested (Table 1) with no infection observed for other *Burkholderia* species or control strains. Sequence analysis revealed a double-stranded DNA genome of 37,727 bp in length with G+C content of 64.5% and 52 predicted coding DNA sequences (see Table S1 in the supplemental material). Due to the presence of an integrase (*gp50*), phiE094 is expected to be a temperate phage. As E094 shares high overall nucleotide sequence identity with other *Burkholderia-*targeting peduoviruses, e.g., phiX216 (96%, JX681814.1), the phage was classified as a member of the *Peduovirinae* subfamily within the *Myoviridae* family, which corresponded with the identification of contractile tails on phage particles by transmission electron microscopy TEM (Fig. 1).

**TABLE 1:** Host range analysis of *Burkholderia* phage E094

Bacterial strain(s)	Spot lysis^*a*^	Plaque formation^*b*^ (% infectivity)
B. pseudomallei (*n* = 8)	5/8 (63%)
1106a	+	+
K96243	+	+
UB1	+	+
UB2	−	−
UB4	+	+
UB5	+	+
UB8	−	−
UB10	−	−
B. thailandensis (*n* = 14)	14/14 (100%)
DV1	+	+
DV2	+	+
DW503	+	+
E027	+	+
E152	+	+
E153	+	+
E192	+	+
E201	+	+
E202	+	+
E207	+	+
E264	+	+
E438	+	+
E440	+	+
E426	+	+
Other *Burkholderia* spp. (*n* = 9)^*c*^	0/12 (0)	0/12 (0)
Other Gram-negative bacteria (*n* = 37)^*d*^	0/37 (0)	0/37 (0)
Other Gram-positive bacteria (*n* = 9)^*e*^	0/9 (0)	0/9 (0)

a Spot lysis: –, no lysis observed; +, lysis comparable to that of B. thailandensis DV1 host stain.

b Plaque formation: –, no plaques observed; +, plaques observed.

c Relative *Burkholderia* species tested: B. cepacia (*n* = 4), B. cepacia complex (*n* = 1), B. oklahomensis (*n* = 1), B. vietnamiensis (*n* = 1), B. ubonensis (*n* = 1), B. multivorans (*n* = 1).

d Other Gram-negative bacteria: Escherichia coli (*n* = 6), Acinetobacter baumannii (*n* = 10), Klebsiella pneumoniae (*n* = 10), Salmonella enterica serovar Typhimurium (*n* = 5), Pseudomonas aeruginosa (*n* = 5), Ralstonia solanacearum (*n* = 1).

e Other Gram-positive bacteria: Staphylococcus aureus (*n* = 5), Listeria monocytogenes (*n* = 4).

### Identification of the tail fiber (gp23) and chaperone (gp24).

**FIG 2:**
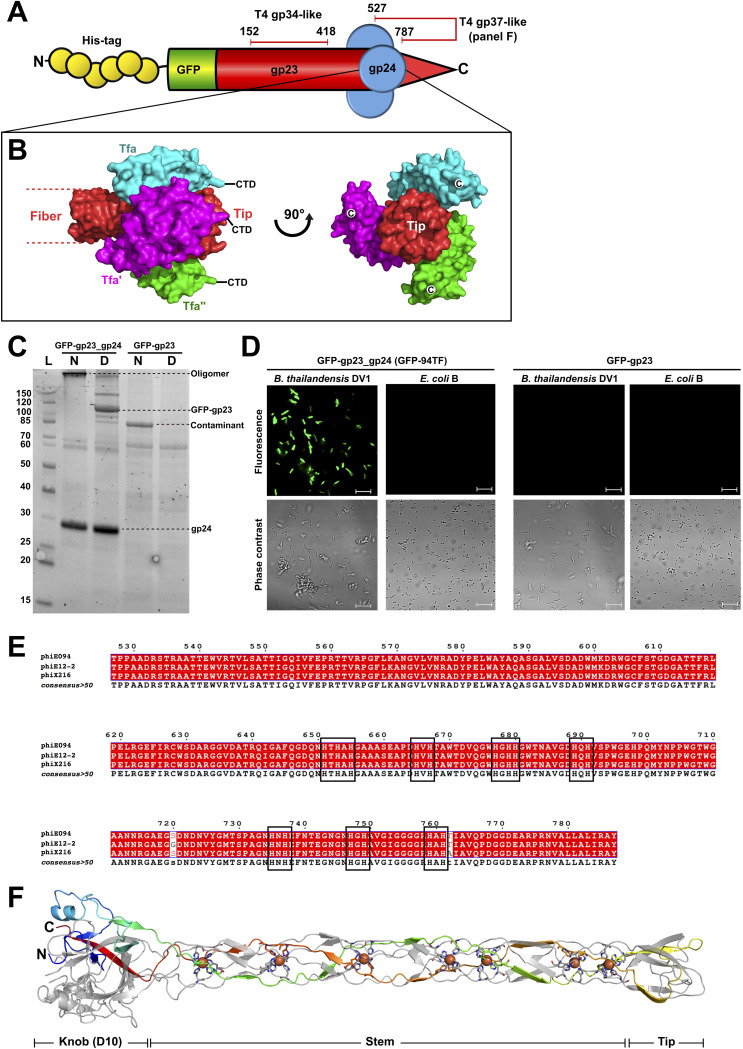
Characterization of the E094 tail fiber (gp23) and assembly protein (gp24) complex (E094TF). (A) Schematic representation of the 94TF bio-probe featuring His and GFP tags on the N-terminus of gp23, which forms a homotrimeric fiber (red) with structural similarity to parts of the proximal (gp34) (49) and distal (gp37) (25) segments of phage T4 LTF, respectively. (B) Surface representation of the three Tfa monomers (cyan, green, magenta) complexed to individual Tfib chains of the phage Mu fiber (red) (PDB ID: 5YVQ) (29) that is predicted to resemble the complex formed between gp23 and gp24 at the tip of 94TF. (C) SDS-PAGE of Ni-NTA purified GFP-94TF with or without gp24 coexpression. Samples were loaded directly (native [N]) or after denaturing for 8 min at 96°C (denatured [D]). Gp24 appeared at a higher molecular weight than expected; however, its correct size was confirmed by LC-MS analysis. Furthermore, the only discernible protein species present after GFP-gp23 purification was identified as an E. coli catalase (KatE) contaminant (see Fig. S1 in the supplemental material). (D) Binding ability of GFP-gp23_gp24 and GFP-gp23 was assessed using fluorescence microscopy against B. thailandensis DV1 and E. coli B. (E) Sequence alignment of the C-terminal region of gp23 (Thr527-Tyr787) with tail fibers of related *Burkholderia* phages phiE12-2 and phiX216 created using ESPript (version 3.0). Conserved residues are shown as white letters on a red background (strict conservation). Black boxes indicate the HXH motifs found within the tail fiber tip and predicted for iron coordination along the length of the fiber. (F) Ribbon representation of the T4 gp37 LTF tip (PDB ID: 2XGF) (25) with individual chains colored cyan, green, and magenta with HXH motif residues shown as sticks and coordinated with iron atoms (orange spheres).

The ability of phage E094 to infect B. pseudomallei and B. thailandensis strains implied that its RBP must possess a similar or broader binding range, making it an interesting candidate for B. pseudomallei detection. Bioinformatics analyses identified gp23 as a putative tail fiber (Fig. 1C) with HHpred identifying structural similarity between its C-terminus and the tip of the Escherichia coli phage T4 long tail fiber (gp37) (25). Gp37 is a homotrimeric fiber that recognizes OmpC and LPS on the bacterial surface (26). Each gp37 chain features HXH motifs (25) that coordinate Fe^2+^ ions upon trimerization. Seven HXH motifs are present within the C-terminus of gp23 and related *Burkholderia* phage fibers (Fig. 2E and F), suggesting a similar trimeric configuration to that of T4 gp37.

As many phages feature downstream chaperones (27–29) for folding or to bestow binding functionality to tail fibers, we assumed that gp24 functioned similarly. HHpred predicted similarity between gp24 and the tail fiber assembly protein (Tfa_Mu_) of phage Mu. Tfa_Mu_ coexpression is required for production of the Mu fiber (Tfib_Mu_), and Tfa_Mu_ remains bound to the Tfib_Mu_ tip after fiber maturation (29, 30) and assists with LPS binding (31) (Fig. 2A). To test if gp24 functioned like Tfa_Mu_, green fluorescent protein (GFP)-tagged gp23 was produced with or without coexpression of gp24 and its ability to bind *Burkholderia* was assessed (Fig. 2C and D). As expected, GFP-gp23 was only produced after gp24 coexpression (GFP-gp23_gp24). Without gp24 coexpression there was no visible GFP-gp23 production and no fluorescence observed. Furthermore, the copurification of gp24 after coexpression indicated that gp24 must interact with GFP-gp23 during Ni-nitrilotriacetic acid (Ni-NTA) purification, likely via formation of a complex similar to that of the phage Mu fiber (29, 30). SDS-resistant GFP-gp23 oligomers in the non-heat-treated GFP-gp23_gp24 sample suggested that gp23 forms a robust fiber complex similar to that of other phage RBPs (32, 33) (Fig. 2C). Meanwhile, the release of gp24 in both heated and nonheated samples suggested that a weaker, SDS-susceptible interaction exists between gp24 and gp23. Fluorescence microscopy showed that GFP-gp23_gp24 was capable of binding to B. thailandensis DV1 with no binding observed against an E. coli B control. As expected, GFP-gp23 produced alone did not demonstrate any binding (Fig. 2D). Clearly, the GFP-gp23 fiber is only functional and capable of host interaction when coexpressed with gp24. During assay development and analysis, the recombinant fiber (GFP-gp23_gp24) was termed GFP-94TF.

**FIG 3:**
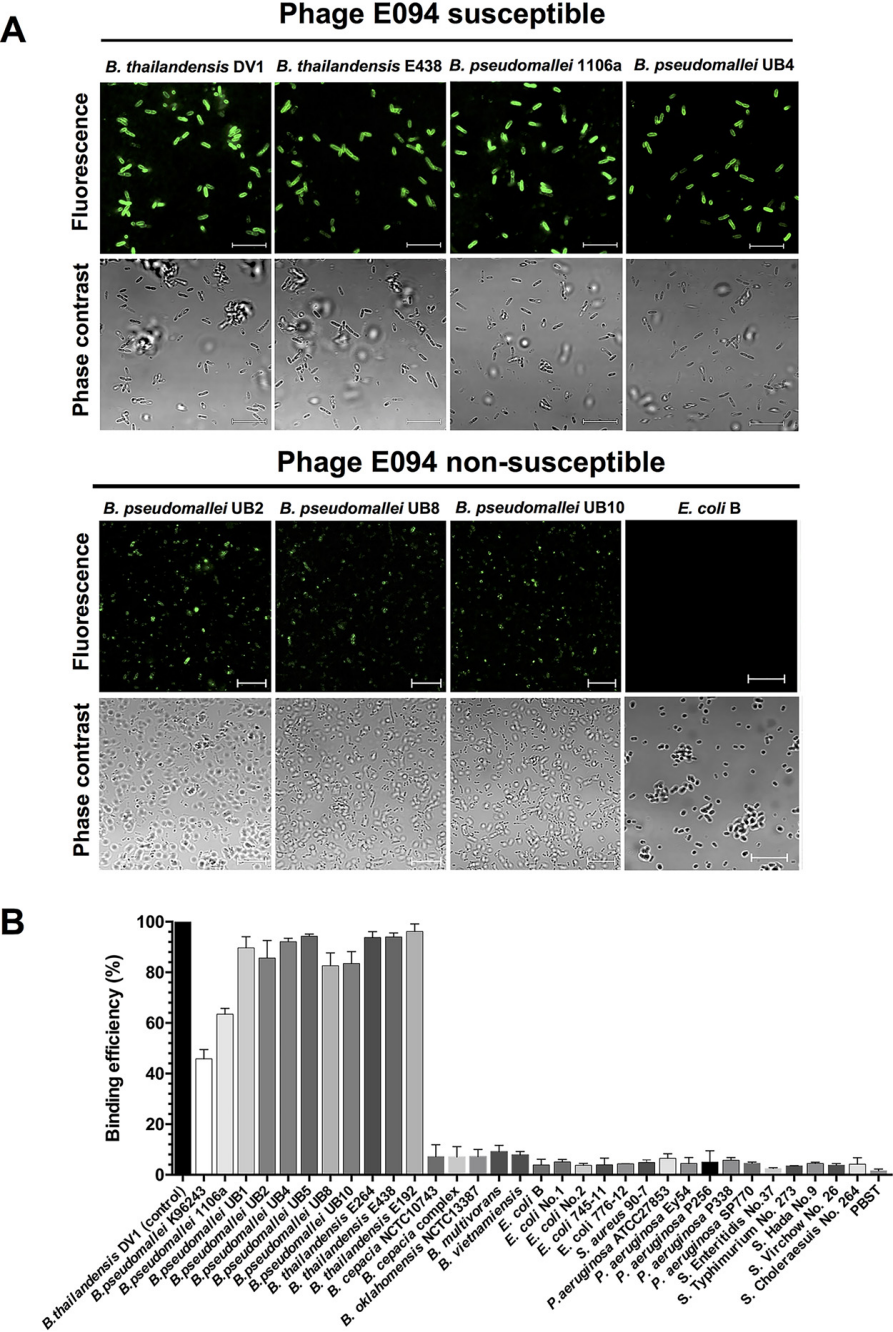
Host binding range analysis of GFP-tagged 94TF. (A) Representative confocal fluorescence micrographs of GFP-94TF binding to phage susceptible B. thailandensis strains DV1 and E438 and B. pseudomallei strains 1106a and UB4 and to phage nonsusceptible B. pseudomallei strains UB2, UB8, and UB10 and E. coli B. (B) Fluorescence spectrometry measurements of binding efficiency of GFP-94TF against various *Burkholderia* strains, as well as an assortment of non-*Burkholderia* strains and PBS-T buffer alone (control). Binding efficiency (%) was calculated relative to the fluorescence intensity observed with B. thailandensis DV1 and defined as 100%. Results represent the mean ± standard deviation (SD) of triplicate experiments. Scale bars indicate 10 μm.

To investigate the binding range of GFP-94TF, fluorescence microscopy and spectrometry were performed against various *Burkholderia* strains and control bacteria. GFP-94TF demonstrated binding to all 10 B. pseudomallei and 34 B. thailandensis strains tested (Table 2), including three B. pseudomallei strains, UB2, UB8, and UB10, that were resistant to phage E094 infection (Fig. 3A). Interestingly, GFP-94TF did not evenly decorate these strains (as observed for phage susceptible hosts), suggesting strain-to-strain binding variability. No binding was observed for other *Burkholderia* species or control strains, which was promising for the further development of GFP-94TF as a B. pseudomallei bio-probe.

**TABLE 2:** Comparative analysis of GFP-94TF binding and 94TF-LAA identification of B. pseudomallei^*a*^

Bacterial species (*n*) and sample type	Protein binding results		94TF-LAA results	
	No. tested	No. positive GFP-94TF binding	No. tested	No. positive agglutination
B. pseudomallei (*n* = 105)
Clinical isolates including blood, pus, lung, sputum, urine, etc.	4	4	85	85
Environment	6	6	10	10
B. thailandensis (*n* = 34)
Environment	34	34	34	34
Total	44	44	129	129
Other bacteria (*n* = 174) (human, animal, and environment)
B. cepacia	4	0	4	0
B. cepacia complex	1	0	1	0
B. multivorans	1	0	1	0
B. oklahomensis	1	0	1	0
B. vietnamiensis	1	0	1	0
Acinetobacter baumannii	38	0	38	3
*Salmonella* spp.	32	0	32	0
*Shigella* spp.	4	0	4	0
Escherichia coli	12	0	12	0
Pseudomonas aeruginosa	19	0	19	0
Staphylococcus aureus	45	0	45	0
Listeria monocytogenes	15	0	15	0
Ralstonia solanacearum	1	0	1	0
Total	174	0	174	3

a 94TF-LAA, phage E094 tail fiber-based latex agglutination assay; GFP-94TF, green fluorescence-tagged E094 tail fiber protein.

### GFP-94TF functions at different ionic strengths, pHs, and temperatures.

**FIG 4:**
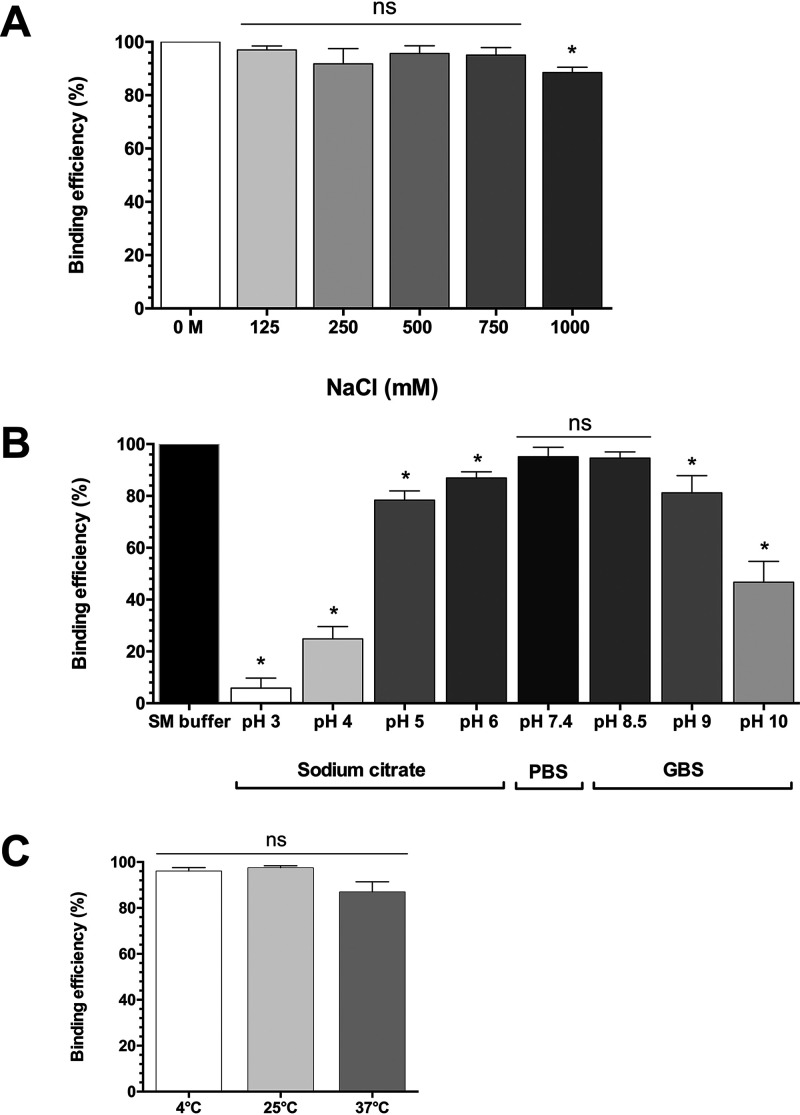
The stability of GFP-94TF when stored at various salt concentrations, pHs, and temperatures was assessed as retained binding efficiency. The binding of GFP-tagged 94TF to B. thailandensis DV1 was assessed at different ionic strengths (A) (NaCl concentration range 0 to 1 M), pHs (B) (pH 3 to 10), and temperatures (C) (4, 25, or 37°C). Cell binding in SM buffer was used as a positive control. Binding efficiency (%) was normalized against the fluorescence intensity observed in SM buffer, which also served as a positive control. Results represent the mean ± standard deviation (SD) of triplicate experiments. Asterisk indicates statistical significance (*P *≤ 0.05, *t* test) when comparing binding efficiency with the controls under the same conditions. ns, no statistical significance.

Due to the variability of clinical specimen composition, bio-probes used for bacterial diagnostics should be robust and retain binding specificity over a range of temperatures, pHs, and salt concentrations (34). We tested the ability of GFP-94TF to bind B. thailandensis DV1 at different NaCl concentrations (0 to 1 M; pH 7.4), buffer conditions (pH 3 to 10), and temperatures (4, 25, and 37°C) (Fig. 4). Optimal binding was observed in phosphate buffer saline (PBS; pH 7.5; 95%) or glycine buffer saline (GBS; pH 8.5; 94%), with the efficiency in binding dropping in increasingly acidic or basic conditions (pH 3: 5.8%; pH 10: 45% binding). Different salt concentrations and temperatures had a negligible effect on binding efficiency. As GBS, pH 8.5 is recommended when using polystyrene latex beads (35), and as it did not affect binding, this buffer was selected for latex agglutination assay development.

### Development of the GFP-94TF latex agglutination assay (94TF-LAA).

**FIG 5:**
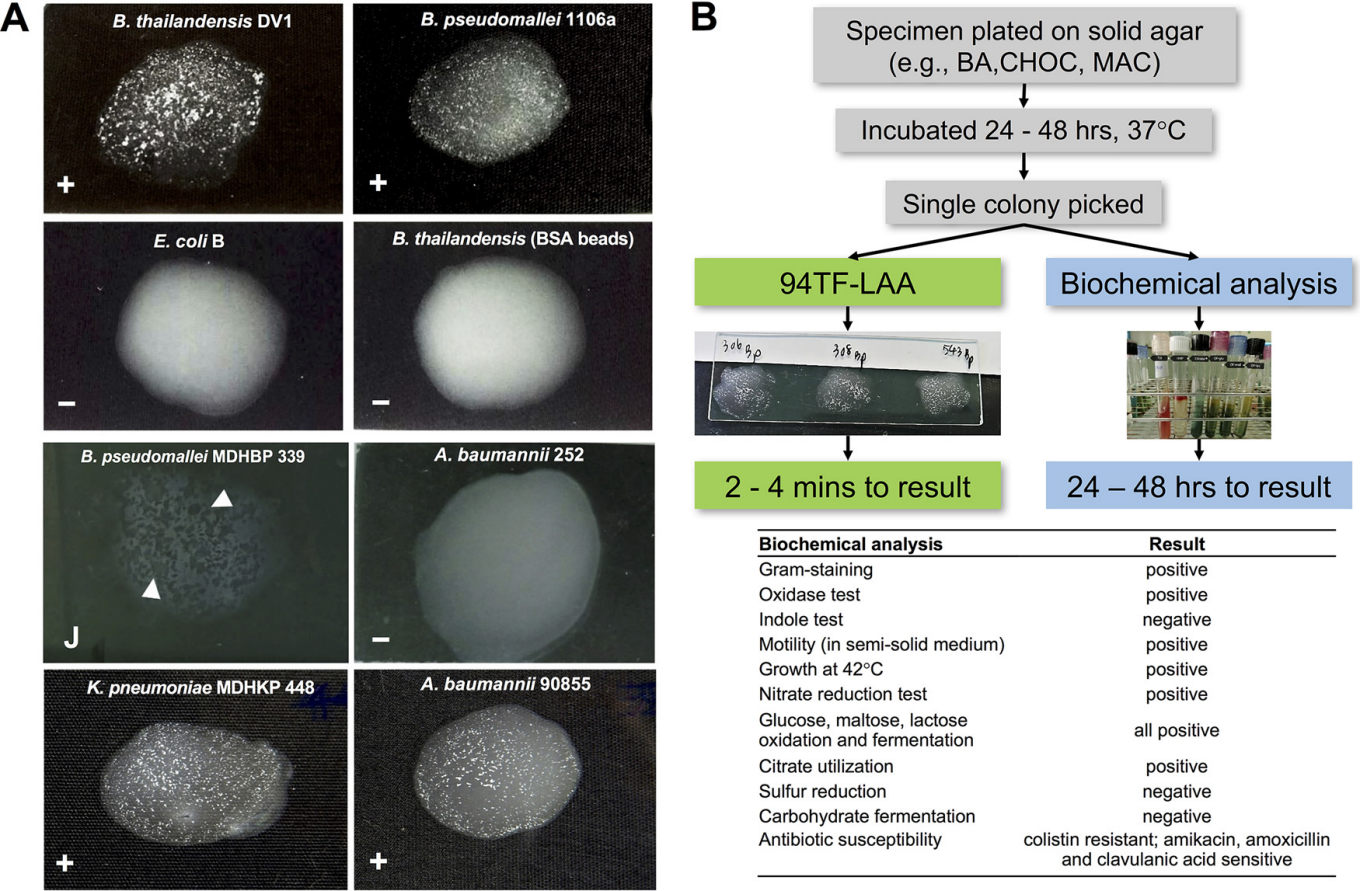
Detection of B. pseudomallei using the 94TF-LAA. (A) Representative results are shown for positive agglutination (+; B. thailandensis DV1 and B. pseudomallei 1106a), negative agglutination (−; E. coli B and A. baumannii 252 and B. thailandensis DV1 reacting with BSA-coated beads), jelly-like clumping (J; B. pseudomallei MDHBP339), and false-positive clinical samples (+; K. pneumoniae MDHKP448 and A. baumannii 90855). (B) Schematic of the field evaluation involving parallel testing of clinical specimens by biochemical analysis and the 94TF-LAA. BA, blood agar; CHOC, chocolate agar; MAC, MacConkey agar.

Optimal assay conditions were first established for using GFP-94TF in an LAA. Increasing amounts of GFP-94TF (50 to 450 μg/ml) were conjugated to various amounts of latex beads (0.5 to 2% wt/vol) in GBS (pH 8.5) containing 1% bovine serum albumin (BSA), and the ability of 10 μl beads to agglutinate B. thailandensis DV1 was assessed. Agglutination was most prominent with GFP-94TF concentrations of 150 to 200 μg/ml and bead concentrations of 0.6 to 0.8%. Importantly, GFP-94TF conjugated beads did not spontaneously agglutinate without bacteria present. The final conditions chosen were 0.6% latex beads coated with 150 μg/ml GFP-94TF stored in GBS (+1% BSA; pH 8.5). Under these conditions, beads clumped as expected within 2 to 4 min when mixed with a bacterial colony (Fig. 5A). No agglutination occurred for the control strain E. coli B or for B. thailandensis DV1 mixed with BSA-coated (empty) beads, with both controls maintaining a milky appearance after 5 min incubation.

The 94TF-LAA was tested against 95 B. pseudomallei strains, including the 10 strains that demonstrated positive binding by the GFP-94TF protein alone (Fig. 3). Remarkably, all 95 isolates produced positive agglutination (Table 2), as well as the 34 B. thailandensis isolates tested. The assay specificity was tested using different *Burkholderia* species and other bacteria (*n* = 174). Only three Acinetobacter baumannii isolates (153, 75642, and 90855) produced agglutination (Fig. 5A), which was not observed for BSA-coated beads (Fig. S2 in the supplemental material). Interestingly, no GFP-94TF binding was observed by fluorescence spectrometry or microscopy against these strains (Fig. S3 in the supplemental material). Potentially, GFP-94TF produces low affinity binding for a cell wall component of these strains that is too weak to detect by fluorescence; however, due to the presence of multiple 94TF molecules on latex beads, the accumulated strength (avidity) of this weak interaction produces agglutination.

### Field evaluation of the 94TF-based latex agglutination assay.

The 94TF-LAA was compared to biochemical-based detection using 300 clinical specimens (e.g., hemoculture, pus, sputum, and urine) taken between September 2019 and July 2020 at the Department of Medical Technology and Clinical Pathology, Mukdahan Hospital, Thailand. Following standard procedures, bacteria were first isolated and grown on blood agar. The 94TF-LAA was performed in parallel with conventional biochemical testing by the clinic (Fig. 5B). The results of both biochemical testing and 94TF-LAA were treated dichotomously (positive or negative detection) with the results of biochemical testing used as the reference standard (Table 3). Biochemical analyses identified 50% of all specimens (150/300) as positive for B. pseudomallei, while the 94TF-LAA identified only 147. The three false-negative isolates (confirmed as B. pseudomallei by MALDI-TOF MS) did generate a response from 94TF-coated beads; however, this consisted of jelly-like clumping of the beads, which was difficult to interpret and so was classed as negative (e.g., B. pseudomallei MDHBP339 [Fig. 5A]). Meanwhile, 94TF-LAA correctly identified 82.6% of isolates as not B. pseudomallei. The remaining 26 false-positive isolates consisted of 1 Stenotrophomonas maltophilia, 10 A. baumannii, and 15 Klebsiella pneumoniae isolates. Phage E094 was shown to not infect any of these isolates; however, fluorescence binding was observed for GFP-94TF against two isolates: K. pneumoniae MDHKP563 and A. baumannii MDHAB558 (Fig. S4 in the supplemental material).

**TABLE 3:** Field evaluation of 94TF-LAA’s ability to identify B. pseudomallei compared to that of conventional biochemical analysis

94TF-LAA results^*a*^	Biochemical analysis results (reference)		Total
	B. pseudomallei	Non-B. pseudomallei	
Positive	147	26	173
Negative	3^*b*^	124	127
Total	150	150	300

*^a^*94TF-LAA, E094 tail fiber-based latex agglutination assay.

b Produced jelly-like clumping of beads determined not to be positive agglutination (e.g., B. pseudomallei isolate MDHBP339 [Fig. 5A]).

Recently, we have seen that the nonspecific agglutination observed for over 60% (16/25) of false-positive samples could be reverted to produce no agglutination by using PBS containing 3% (wt/vol) PEG8000 and 0.02% NaN_3_ as the bead storage buffer. While the buffer should be further optimized, this clearly indicates that the specificity of the 94TF-LAA could be improved by assessing other additives and buffers known to generally improve LAA results (35).

During the field evaluation, the 94TF-LAA produced an overall diagnostic sensitivity of 98% (95% CI of 94.27 to 99.59), specificity of 82.7% (95% CI of 75.64 to 88.35), and accuracy of 90.33% (95% CI of 86.41 to 93.43). The assay took less than 5 min from direct colony picking, enabling rapid detection of B. pseudomallei while remaining quicker and easier to perform than conventional biochemical testing. With further characterization of tail fibers from phages isolated on selective *Burkholderia* species it should be possible to produce a broad library of tail fiber affinity molecules for individual *Burkholderia* species identification.

## DISCUSSION

Rapid diagnosis is of critical importance for treatment of melioidosis (4). Considering that over 50% of patients with acute septicemic melioidosis die within 48 h of admission (1, 4), an early diagnosis ensures optimal antimicrobial treatment. In this study, we developed a diagnostic LAA for detection of B. pseudomallei that exploits the specific binding ability of the phage E094 tail fiber. The fiber (gp23) plus tail assembly protein (gp24) architecture of 94TF appears to be conserved among other members of the *Peduovirinae*, including 57% (8/14) of other *Burkholderia* phages (Table S2 in the supplemental material), all of which feature highest sequence plasticity within their C-termini where receptor binding sites are likely located (26). Provided that E094 infects only B. pseudomallei and B. thailandensis, these two species must feature a shared, yet unknown, receptor on their surfaces that is not present on other *Burkholderia* spp.

Antibody-based LAAs are readily available for recognition of capsular polysaccharides (CPS) of B. pseudomallei and B. mallei but not those of B. thailandensis (9, 14, 36), such as the CPS-specific monoclonal antibody 4B11 with separately reported sensitivity and specificity above 99% and 97%, respectively (14, 36). While our 94TF-LAA also generated reasonable levels of specificity (82.7%), this could be improved with assay optimization. In addition, the fact that E094 was originally isolated using a B. thailandensis host meant its tail fiber will always be liable to produce cross-reactivity against this nontarget species. However, given that B. thailandensis infections are rarely reported (37–39), such cross-reactivity is likely not to be an issue when detecting B. pseudomallei using the 94TF-LAA. In any case, as treatment regimens for B. pseudomallei and B. thailandensis infections are analogous (40), a positive result from the 94TF-LAA would still ensure that an optimal antibiotic course is quickly prescribed to patients. Additionally, B. thailandensis can be differentiated from B. pseudomallei due to its ability to assimilate arabinose (41), meaning that a positive result from a 94TF-LAA could be further investigated using biochemical-based detection if required. Potentially, a species-specific phage and its encoded RBP could be identified by using only B. pseudomallei strains for phage isolation; however, this would be extremely difficult given the constraints of working with biosafety level three organisms.

Nevertheless, whether using phage RBPs or antibodies, complete elimination of cross-reactivity is an extremely difficult task, especially for B. pseudomallei detection (14, 36, 42, 43). Similarly, the field evaluation of 94TF-LAA produced 26 false-positive results, including A. baumannii. Cross-reactivity has been observed before with phage-based LAAs, especially when identifying between species within the same genera (44). Misidentification of B. pseudomallei as Acinetobacter spp. has also been observed for antibody-based LAAs (45), and as such, supplementary detection methods should be considered when LAAs are used in clinical diagnostics (1, 4). As bio-probes, phage RBPs use different mechanisms to interact with their host receptors (46), allowing them to bypass various drawbacks of using antibodies as affinity proteins (47). Phage RBPs are also robust and easy to produce as recombinant proteins and are capable of long periods of storage without loss of activity. For example, GFP-94TF was functional after 12 months of storage at 4°C. To the best of our knowledge, this is the first study to use a phage tail fiber for detection of B. pseudomallei. As an easy-to-use diagnostic, the 94TF-LAA has the potential to sit alongside conventional diagnostics in Thai clinics to aid rapid identification of this clinically important pathogen.

## MATERIALS AND METHODS

### Ethical approval.

B. pseudomallei isolates were grown and tested under biosafety level 3 (BSL-3) containment procedures at the Department of Microbiology and Immunology, Faculty of Tropical Medicine, Mahidol University with approval from the Mahidol University Biosafety Committee (approval number MU-IBC 2019-0037) and Biosafety Committee of Khon Kaen University (approval number IBCKKU 1/2563). Culturing of non-B. pseudomallei isolates was approved by the Siriraj Safety Risk Management Taskforce of Mahidol University (approval number SI 2020-002). Field evaluation of 94TF-LAA was approved by the Ethics Committee for Human Research of Mukdahan Hospital (approval number MEC 07/62).

### Phage isolation, purification, and propagation.

*Burkholderia* phages were isolated from soil collected from rice paddy fields in the Ubon Ratchathani Province, northeast Thailand (region of endemicity for melioidosis) as described previously (18). In brief, 5 g of soil was resuspended in 10 ml saline magnesium buffer (SM; containing 5.5 g/liter NaCl, 2 g/liter magnesium sulfate, 1.0 M Tris-HCl [pH 7.4]) plus 5 mM CaCl_2_ and vigorously mixed before separating the supernatant by filtering through a 0.45-μm filter (GE Healthcare, USA). The supernatant was spiked with 5 ml of mid-log B. pseudomallei 1106a, B. thailandensis DV1, and B. thailandensis D1 and incubated with shaking at 37°C for 24 h. The mixture was centrifuged (4,500 × *g*, 20 min) and was collected again by filtration. Phage lysis was identified from each filtrate by a spot assay using B. thailandensis DV1 as a host, with the presence of plaque-forming phages confirmed by double agar overlay plaque assay which was also used to pick and purify individual phages at least eight times. To produce high titer phage stocks, phages were concentrated from lysates using polyethylene glycol (PEG; Sigma-Aldrich, USA) precipitation. Precipitated phages were collected by centrifugation (11,000 × *g*, 30 min at 4°C) and resuspended in 1 ml SM buffer to final titers of ∼10^9^ PFU/ml.

### Genome sequencing, assembly, and annotation.

Phenol chloroform extraction and isopropanol precipitation were used for phage genomic DNA extraction (48). Sequencing was performed using 314v2 chips on Ion Torrent PGM (Thermo Fisher Scientific, USA). The sequenced high-quality reads were *de novo* assembled using CLC Genomics Workbench version 6.0.5 (Qiagen, CA, USA). The CAP3 assembly program was used for construction of the phage contig and removal of false overlaps. Protein coding sequences (CDSs) were identified using the Rapid Annotation using Subsystem Technology tool kit (RASTtk) pipeline. Subsequent manual curation and validation were performed using genomes from related *Burkholderia* phages phiE202 (NC_009234) and phiE12-2 (NC_009236) as references. The genome of *Burkholderia* virus phiE094 was submitted to BankIt NCBI. A final genome map was generated and visualized using Artemis version 18.1.0.

### Phage morphology examination by transmission electron microscopy.

A high titer (10^9^ PFU/ml) phage solution was dropped onto a 100-mesh copper grid coated with Formvar (Ted Pella, Redding, CA, USA) and then negative stained with 2% potassium phosphotungstate (pH 7.2) (Sigma-Aldrich, St. Louis, MO, USA). The grid was examined using a TEM-1230 transmission electron microscope (JEOL, Tokyo, Japan) equipped with a dual vision digital camera (Gatan, Pleasanton, CA, USA).

### Protein construction, expression, and purification.

A pETDUET-based plasmid featuring N-terminal His and GFP tags (27, 33) was used to generate GFP-gp23 and GFP-gp23_gp24 expression plasmids via Gibson assembly (New England Biolabs, USA) using E094 genomic DNA as the initial template and oligonucleotides listed in Table 4. E. coli XL-1 Blue MRF' (Agilent, USA) was used for cloning. Plasmid sequences were confirmed by Sanger sequencing (Eurofins GATC Biotech) before transformation into E. coli BL21(DE3) (New England BioLabs, USA) for protein expression. For protein expression, cells were grown in selective LB media containing 100 μg/ml ampicillin until log-phase growth (optical density at 600 nm [OD_600_] of 0.6) when expression was induced with 0.5 mM isopropyl-d-thiogalactopyranoside (IPTG; Thermo Scientific, USA) for 16 h growth at 20°C with agitation. Cells were harvested by centrifugation (5,500 × *g*, 15 min). Cells were suspended in phosphate buffer saline containing 0.1% Tween 20 (0.1% PBS-T; pH 8.0) and 5 mM imidazole. The cells were cooled to 4°C and lysed using a Stansted pressure cell homogenizer (Stansted Fluid Power, UK) or by sonication (Sonics Vibra cell, USA). Purification was performed by gravity flow immobilized metal affinity chromatography (IMAC) with low-density Ni-NTA resin (Thermo Scientific, USA). 0.1% PBS-T plus 5 mM imidazole was used as a wash buffer and 0.1% PBS-T (pH 8.0) plus 250 mM imidazole was used for elution buffer. Proteins were dialyzed into 25 mM TRIS (pH 7.4) and stored at 4°C. Proteins were analyzed by SDS-PAGE (Criterion TGX stain-free gel) using PageRuler Unstained Protein Ladder (Thermo Scientific, USA) as a marker and imaged by UV or stained with InstantBlue (Expedeon, USA) using a Gel Doc XR imaging system (Bio-Rad).

**TABLE 4:** Oligonucleotides used in this study

Name	Sequence (5′ → 3′)
Insert_gp23_gp24_fw	TGG ATG AAC TAT ACA AAG AGC TCA TGC TCA TCA ACA TCA CCG A
Insert_gp23_gp24_bw	CAA GCT TGT CGA CCT GCA GTC AGG CCG GGG CGT
Vector_gp23_gp24_fw	TCG GTG ATG TTG ATG AGC ATG AGC TCT TTG TAT AGT TCA TCC A
Vector_gp23_gp24_bw	ACG CCC CGG CCT GAC TGC AGG TCG ACA AGC TTG
Insert_gp23_fw	AAA GAG CTC ATG CTC ATC AAC AT
Insert_gp23_bw	TTA GTA GGC GCG AAT CA
Vector_gp23_fw	ATG TTG ATG AGC ATG AGC TCT TT
Vector_gp23_bw	GCT GAT TCG CGC CTA CTA ACT GCA GGT CGA CAA GCT TG

### Fluorescence-based analysis of GFP-94TF binding.

Bacterial strains used are listed in Table 5. Bacteria were grown to mid-log phase in LB broth at 37°C for 2 to 4 h. In brief, 50 μg GFP-94TF was mixed with 10^6^ CFU/ml of bacterial cells using an overhead rotator for 30 min at room temperature. Bacteria were spun down (5,000 × *g*, 5 min), washed twice with 1 ml 0.1% PBS-T, and resuspended in 200 μl 0.1% PBS-T in a 96-well Nunc MicroWell polystyrene plate (Sigma-Aldrich, USA). Binding of GFP-94TF was measured using fluorescence spectrometry with a SynergyH1 Hybrid Multi-Mode plate reader (BioTek Instrument, USA). For confocal fluorescence microscopy measurements, 10 μl of cells were dropped onto a glass slide and covered by a cover slip. Laser scanning confocal microscopy (LSM800, Carl Zeiss, Jena, Germany) was performed at ×120 magnification using an oil immersion lens. Images were acquired using an AxioObserver7 microscope camera (Carl Zeiss, Jena, Germany). Binding efficiency (%) is reported as the average fluorescence intensity observed for triplicate measurements normalized against fluorescence intensity observed for the propagation strain B. thailandensis DV1. Binding was performed in triplicate with graphs produced using GraphPad Prism (v6.0) and represented as mean fluorescence ± standard deviation.

**TABLE 5:** Bacterial strains used in this study

Bacterial species	Source/sample type	Strain(s)/other designation(s)
*Burkholderia* spp.		
B. pseudomallei	Clinical/human	K96243, 1106a, UB1, UB2, UB4, UB5, UB8, UB10, DR50020A, DR50021A, DR50022A, DR50023A, DR50024A, DR50025A, DR50026A, DR50027A, DR50028A, DR50029A, DR50030A, DR50031A, DR50032A, DR50033A, DR50034A, DR50035A, DR50036A, DR50037A, DR50038A, DR50039A, DR50040A, DR50041A, DR50042A, DR50043BPA, DR50044BPA, DR50045BPA, DR50046BPA, DR50047BPA, DR50048BPA, DR50049BPA, DR50050BPA, DR50051BPA, DR50052BPA, DR50053BPA, DR50054BPA
	Clinical/blood	H777, EPBR012 (B1), EPBR013 (B2), EPMN099 (B3), EPNP218 (B4), EPBR017, EPBR018, EPBR019, EPBR032, EPBR040, EPMN107, EPMN110, EPMN130, EPMD003, EPMN061, EPNK004, EPNK007
	Clinical/pus	EPCP117 (P1), EPSR155 (P2), EPSR176 (P3), EPNP322 (P4), EPAC023, EPAC048, EPAC049, EPAC053, EPAC055, EPAC065
	Clinical/lung	EPMN092 (L1), EPSR116 (L2), EPSR156 (L3)
	Clinical/sputum	EPBR015, EPNK005, EPNK017, EPNK020, EPNK022, EPNK039, EPNK047, EPNK050, EPNK066, EPNK071
	Clinical/urine	EPNK006, EPNK024, EPNK073, EPNK193, EPNL152, EPNP174, EPNP124, EPRE007, EPRE121
	Clinical/synovial fluid	EPNK107
B. pseudomallei^*a*^	Clinical/hemoculture	MDHBP802, MDHBP1512, MDHBP1868, MDHBP1937, MDHBP413, MDHBP561, MDHBP804, MDHBP861, MDHBP899, MDHBP1081, MDHBP1081, MDHBP1384, MDHBP1449, MDHBP1487, MDHBP250, MDHBP543, MDHBP1092, MDHBP1250, MDHBP1374, MDHBP559, MDHBP88, MDHBP137, MDHBP318, MDHBP326, MDHBP367, MDHBP590, MDHBP603, MDHBP725, MDHBP798, MDHBP949, MDHBP1237, MDHBP1287, MDHBP1538, MDHBP963, MDHBP858, MDHBP1430, MDHBP1448, MDHBP369, MDHBP605, MDHBP880, MDHBP998, MDHBP1054, MDHBP33, MDHBP884, MDHBP904, MDHBP921, MDHBP6, MDHBP767, MDHBP730, MDHBP1295, MDHBP11, MDHBP40, MDHBP94, MDHBP84, MDHBP233, MDHBP402, MDHBP369, MDHBP413, MDHBP541, MDHBP554, MDHBP1571, MDHBP1, MDHBP140, MDHBP174, MDHBP351, MDHBP279, MDHBP296
	Clinical/sputum	MDHBP825, MDHBP60, MDHBP236, MDHBP293, MDHBP658, MDHBP783, MDHBP779, MDHBP54, MDHBP219, MDHBP19, MDHBP197, MDHBP533, MDHBP826, MDHBP136, MDHBP630, MDHBP645, MDHBP402, MDHBP450, MDHBP610, MDHBP876, MDHBP247, MDHBP280, MDHBP867, MDHBP102
	Clinical/pus	MDHBP842, MDHBP75, MDHBP259, MDHBP325, MDHBP336, MDHBP401, MDHBP530, MDHBP481, MDHBP470, MDHBP29, MDHBP23, MDHBP306, MDHBP308, MDHBP357, MDHBP425, MDHBP423, MDHBP562, MDHBP646, MDHBP307, MDHBP347, MDHBP448, MDHBP458, MDHBP490, MDHBP608, MDHBP694, MDHBP742, MDHBP749, MDHBP278, MDHBP700, MDHBP62, MDHBP383, MDHBP524, MDHBP632, MDHBP333, MDHBP404, MDHBP5, MDHBP17, MDHBP37, MDHBP335, MDHBP628, MDHBP112, MDHBP421, MDHBP558, MDHBP1153, MDHBP774, MDHBP897, MDHBP914, MDHBP114, MDHBP173, MDHBP202
	Clinical/urine	MDHBP297, MDHBP339, MDHBP845, MDHBP46, MDHBP552, MDHBP136, MDHBP182
	Clinical/ascites	MDHBP210
	Clinical/synovial fluid	MDHBP806
	Environment	MBPE229, MBPE228, MBPE239, MBPE243, MBPE244, MBPE234, 3E, 8E, 23E, ST39
B. thailandensis	Environment	DV1, DV2, DW503, E27, E264, E152, E153, E154, E158, E159, E169, E173, E174, E175, E177, E201, E202, E205, E192, E207, E243, E246, E352, E360, E421, E426, E427, E430, E433, E435, E436, E438, E440, E441
B. cepacia	Clinical/human	NCTC10743, NCTC10744, NVDh30, NVDh31
B. cepacia complex	Clinical/human	SI01
B. multivorans	Clinical/human	LMG16660
B. oklahomensis	Clinical/human	BOC6786
B. vietnamiensis	Clinical/human	LMG6999
Non-*Burkholderia* spp.		
Acinetobacter baumannii	Clinical/human	No. 9, no. 40, no. 72, no. 131, no. 153, no. 136, no. 184, no. 190, no. 251, no. 252, no. 208, no. 288, 70253-B1, 72770-B2, 72568-B3, 72171-B4, 72956-B5, 72946-B6, 72666-B7, 72957-B8, 73099-D1, 74011-D5, 73810-D6, 74217-D7, 74731-D9, 75442-D10, 75642-E1, 77565-E2, 75568-E4, 75508-E5, 76106-E9, 76484-E10, 90855, 91018, Ac1 (spl), Ac2 (H1), Ac3
Acinetobacter baumannii^*a*^	Clinical/human	MDHAB324, MDHAB358, MDHAB384, MDHAB373, MDHAB386, MDHAB387, MDHAB485, MDHAB506, MDHAB647, MDHAB678, MDHAB694, MDHAB784, MDHAB841, MDHAB15, MDHAB80, MDHAB91, MDHAB117, MDHAB136, MDHAB137, MDHAB158, MDHAB162, MDHAB16, MDHAB196, MDHAB200, MDHAB219, MDHAB235, MDHAB360, MDHAB407, MDHAB494, MDHAB455, MDHAB554, MDHAB558, MDHAB1058, MDHAB567, MDHAB592, MDHAB632, MDHAB663, MDHAB510
Citrobacter diversus^*a*^	Clinical/human	MDHCD600
Citrobacter freundii^*a*^	Clinical/human	MDHCF27, MDHCF442
Chromobacterium violaceum^*a*^	Clinical/human	MDHCV470
Escherichia coli	Clinical/human	No. 1, no. 2, no. 3, no. 4, no. 5, no. 8, no. 745-11, no. 776-12, no. 777-13, no. 785-14, no. 785-15
Escherichia coli^*a*^	Clinical/human	MDHEC362, MDHEC371, MDHEC339, MDHEC378, MDHEC677, MDHEC687, MDHEC696, MDHEC397, MDHEC801, MDHEC856, MDHEC489, MDHEC1021, MDHEC925, MDHEC608, MDHEC611, MDHEC634, MDHEC649, MDHEC731, MDHEC760, MDHEC1021
Enterobacter aerogenes^*a*^	Clinical/human	MDHEA408, MDHEA387, MDHEA701
Enterobacter cloacae^*a*^	Clinical/human	MDHECL72, MDHECL596, MDHECL485
Enterococcus faecalis^*a*^	Clinical/human	MDHEF642
Klebsiella pneumoniae^*a*^	Clinical/human	MDHKP364, MDHKP369, MDHKP371, MDHKP450, MDHKP455, MDHKP478, MDHKP565, MDHKP510, MDHKP536, MDHKP595, MDHKP610, MDHKP626, MDHKP627, MDHKP640, MDHKP666, MDHKP671, MDHKP687, MDHKP845, MDHKP3, MDHKP9, MDHKP88, MDHKP103, MDHKP145, MDHKP151, MDHKP178, MDHKP243, MDHKP249, MDHKP244, MDHKP247, MDHKP252, MDHKP446, MDHKP495, MDHKP368, MDHKP480, MDHKP479, MDHKP448, MDHKP547, MDHKP563, MDHKP583, MDHKP551, MDHKP614, MDHKP672
Pseudomonas aeruginosa^*a*^	Clinical/human	MDHPA320, MDHPA365, MDHPA377, MDHPA442, MDHPA492, MDHPA565, MDHPA508, MDHPA537, MDHPA625, MDHPA824, MDHPA800, MDHPA806, MDHPA813, MDHPA33, MDHPA50, MDHPA325, MDHPA328, MDHPA337, MDHPA443
*Pseudomonas* spp.^*a*^	Clinical/human	MDHP386, MDHP429
Pseudomallei aeruginosa	Animal	No. 34, no. 41, no. 851, no. 881, no. 896, no. 898, no. 900, no. 947, no. 953, no. 958, no. 990, no. 996, no. 1005, no. 1017, no. 1083, no. 1087, no. 1093, no. 1093, no. 1170, no. 1171
Proteus mirabilis^*a*^	Clinical/human	MDHPM556, MDHPM488, MDHPM678, MDHPM734
Proteus vulgaris^*a*^	Clinical/human	MDHPV608
*Salmonella* spp.	Clinical/human	No. 129, no. 130, no. 170, no. 177, no. 181, no. 184, no. 229, no. 262, no. 271, no. 272, no. 275, no. 278, no. 280, no. 282, no. 283, no. 288, no. 289, no. 292, no. 294, no. 295, no. 300, no. 304, no. 308, no. 313, no. 171, no. 264, no. 297, no. 265, no. 290, no. 296, no. 298, no. 304, no. 308, no. 313, DMST, no. 171, no. 264, no. 297, no. 265, no. 290, no. 296, no. 298
*Salmonella* group E^*a*^	Clinical/human	MDHSE67
*Shigella* spp.	Clinical/human	B1, no. 155, no. 789-110, no. 1982-3
Stenotrophomonas maltophilia^*a*^	Clinical/human	MDHSM325, MDHSM455, MDHSM542, MDHSM601, MDHSM648, MDHSM1088, MDHSM684
β-*Streptococcus* group B^*a*^	Clinical/human	MDHSB1154
Staphylococcus aureus^*a*^	Clinical/human	MDHSA1137
Staphylococcus aureus	Clinical/human	No. 1, no. 2, no. 3, no. 4, no. 5, no. 90-7, no. 453-10, no. 532-9, no. 549-8, no. 1457-6, US001, US002, US003, US004, US005, US006, US007, US008, US009, US0010, US0011, US0012, US0013, US0014, US0015, US0016, US0017, US0018, US0019, US0020, US0021, US0022, US0023, US0024, US0025, US0026, US0027, US0028, US0029, US0030, US0031, US0032
Listeria monocytogenes	Clinical/human	5509-9, 4960-8, 4961-8, 4852-9I, 4853-9, 3632-3, 3649-9I, 1401-9, 1717-7HC, 4275I-2, 4331-7, 1401-9, 1225-3I

a Isolates were collected from clinical samples (Mukdahan hospital) used for field evaluation of 94TF-LAA.

### Binding efficiency testing in different buffer compositions.

Five hundred microliters of a mid-log culture of B. thailandensis DV1 (1 × 10^6^ CFU/ml) was pelleted and resuspended in different NaCl concentrations (0 to 1 M; pH 7.4) or pHs (buffer citrate, pH 3 to 6; PBS, pH 7.4; GBS [100 mM glycine buffer solution and 100 mM NaCl], pH 8.5 to 10) and stored at 4, 25, or 37°C. Purified GFP-94TF (50 μg) was added to the B. thailandensis DV1 cells and stored for 30 min. Cells were pelleted and washed twice in 1 ml 0.1% PBS-T. Binding of GFP-94TF was measured using fluorescence spectrometry and reported as described above.

### Direct colony latex agglutination assay (94TF-LAA).

Five hundred microliters of 2% polystyrene sulfate latex beads (0.8 μm diameter; Thermo Fisher Scientific, USA) were washed 3 times and resuspended to the same volume in 100 mM GBS (pH 8.5) using centrifugation (16,000 × *g*, 5 min) to separate beads between wash steps. Ni-NTA purified GFP-94TF (150 μg/ml) was mixed with the beads for 16 h at 4°C. The GFP-94TF-conjugated beads were pelleted (16,000 × *g*, 5 min) and then blocked three time with 1 ml of 100 mM GBS buffer containing 1% (wt/vol) bovine serum albumin (BSA; Sigma-Aldrich, USA) on an orbital shaker for 30 min at room temperature. Beads were washed and resuspended in the same solution at a final concentration of 0.6% bead suspension. The beads were stored at 4°C and brought to room temperature before use. BSA-coated beads were produced following the same steps (by exchanging GFP-94TF for 1% BSA) and subsequently used as a negative control.

To perform the LAA, 10 μl of GFP-94TF (or BSA) coated beads were dropped on a clean glass slide and mixed with a single bacterial colony picked from a culture grown on MacConkey agar (Oxoid, UK). For field evaluation, the same colony was used for both 94TF-LAA and biochemical-based detection. The slide was rotated gently, and results were read after 2 to 4 min. Positive agglutination (e.g., B. pseudomallei 1106a) was observed as large clumps, while negative agglutination (e.g., E. coli B) remained a milky solution. Results were treated dichotomously (positive or negative for B. pseudomallei) with biochemical testing used as the reference standard.

### Biochemical-based detection of B. pseudomallei.

Biochemical-based detection was performed according to the ASM Clinical Laboratory Guidelines as recommended (4) and following ISO/IEC 17025 guidelines. The detailed procedure of biochemical identification and antimicrobial susceptibility tests is described in the supplemental material.

### Mass spectrometry analysis of protein species.

Protein species in solution were identified using liquid chromatography-electrospray ionization-mass spectrometry (LC-ESI-MS), and SDS-PAGE purified gel bands were identified by liquid chromatography MS/MS (LC-MS-MS). All analyses were performed by the Functional Genomics Center Zurich, Switzerland as described in the supplemental material.

### Data availability.

The genome sequence and associated data for *Burkholderia* phage phiE094 were deposited under GenBank accession number MW072790.
