# Supplementary material for: Rapid Clinical Screening of Burkholderia pseudomallei Colonies by a Bacteriophage Tail Fiber-Based Latex Agglutination Assay
Source: Appl Environ Microbiol. 2021 May 26;87(12):e03019-20. doi: 10.1128/AEM.03019-20 (PMC8174754; doi:10.1128/AEM.03019-20)
Supplement: SUPPLEMENTAL FILE 1 — Supplemental material. Download aem.03019-20-s0001.pdf, PDF file, 1.1 MB [file aem.03019-20-s0001.pdf]

SUPPLEMENTAL MATERIAL

Supplemental material is available online only.

TABLE S1, DOCX file, 0.02 MB.

TABLE S2, DOCX file, 0.03 MB.

FIG S1, TIF file, 4.6 MB.

FIG S2, TIF file, 5.6 MB.

FIG S3, TIF file, 3 MB.

FIG S4, TIF file, 6 MB.

Supplemental material, DOCX file, 0.02 MB.

TABLE S1 Feature table of 52 predicted CDSs in *Burkholderia* virus phiE094. Highlighted in bold are the tail fiber (gp23) and tail fiber chaperone (gp24)

CDSs	Start (nt)	Stop (nt)	Strand	Length (bp)	Predicted function
1	101	790	+	690	Phage terminase, small subunit
2	791	1105	+	315	Putative toxin protein, addiction module killer protein
3	1108	1464	+	357	Putative antitoxin protein, module antidote protein
4	2563	1508	-	1056	Phage portal vertex protein
5	4329	2560	-	1770	Phage terminase, ATPase subunit
6	4473	5282	+	810	Phage capsid scaffolding protein
7	5316	6329	+	1014	Phage major capsid protein
8	6412	6645	+	234	Phage head completion-stabilization protein
9	6645	6896	+	252	hypothetical bacteriophage protein
10	6893	7099	+	207	Phage tail protein
11	7114	7458	+	345	Phage holin
12	7460	7732	+	273	Phage membrane protein
13	7729	8541	+	813	Putative phage-encoded peptidoglycan binding protein, endolysin
14	8538	8978	+	441	Phage LysB protein
15	8932	9090	+	159	Phage LysC protein
16	9083	9499	+	417	Phage P2 tail completion R-like protein
17	9496	9963	+	468	Phage P2 tail completion S-like protein
18	11190	10363	-	828	DNA methytransferase
19	11295	11975	+	681	Phage baseplate assembly V-like protein
20	11972	12334	+	363	Phage baseplate assembly W-like protein
21	12331	13236	+	906	Phage baseplate assembly J-like protein
22	13229	13783	+	555	Phage tail formation protein
**23**	**13785**	**16157**	**+**	**2373**	**Phage tail fiber protein**
**24**	**16174**	**16845**	**+**	**672**	**Phage tail fiber assembly chaperone**
25	16901	18073	+	1173	Phage tail sheath monomer
26	18089	18598	+	510	Phage major tail tube protein
27	18656	18997	+	342	Phage tail assembly protein
28	19006	19119	+	114	Phage P2 GpE family protein
29	19122	22241	+	3120	Phage tail length tape-measure protein
30	22315	22524	+	210	Phage tail protein

TABLE S1 Feature table of 52 predicted CDSs in *Burkholderia* virus phiE094. (cont.)

CDSs	Start (nt)	Stop (nt)	Strand	Length (bp)	Predicted function
31	22524	23621	+	1098	Phage tail formation D-like protein
32	24528	23629	-	900	Phage protein
33	25311	24568	-	744	Alpha/ beta hydrolase
34	25792	25361	-	432	Cro/CI family transcriptional regulator
35	25917	26111	+	195	Phage protein
36	26115	26411	+	297	DNA-binding protein
37	26399	26647	+	249	Ogr/Delta-like zinc finger family transcriptional activator
38	26732	26950	+	219	Hypothetical protein
39	26956	27153	+	198	Hypothetical protein
40	27196	27390	+	195	Hypothetical protein
41	27394	27633	+	240	Hypothetical protein
42	27802	28008	+	207	Phage protein
43	28011	28370	+	360	Hypothetical protein
44	28367	28621	+	255	DNA primase
45	28633	31425	+	2793	Topoisomerase-primase, DNA segregation ATPase FtsK/SpoIIIE and related proteins
46	32031	31810	-	222	Hypothetical protein
47	33763	32051	-	1713	Cytidine and deoxycytidylate deaminase, zinc-binding region family protein
48	34600	33788	-	813	AAA family ATPase
49	35400	34597	-	804	AAA family ATPase
50	36964	35855	-	1110	Phage integrase
51	37120	37329	+	210	Hypothetical protein
52	37518	37727	+	210	Phage protein

TABLE S2 BLASTp analysis of tail fiber (gp23) and chaperone (gp24) among *Burkholderia* phage homologs

Phage name	Bacterial host	GenBank Accession no.	% Protein sequence similarity (sequence ID)	
			phiE094 gp23	phiE094 gp24
Burkholderia phages E12-2	*B. pseudomallei*	NC_009236.1	99.1% (YP_001111185)	99.55% (YP_001111184.1)
Burkholderia phages X216	*B. pseudomallei*	JX681814	98.6% (AFV51415.1)	99.55% (AFV51416.1)
Burkholderia phages E202	*B. thailandensis*	NC_009234.1	98.0% (YP_001111063.1)	98.21% (YP_001111062.1)
Burkholderia phages KS5	*B. cepacia* complex	NC_015265.1	77.5% (YP_004306390.1)	58.73% (YP_004306389.1)
Burkholderia phages KL3	*B. cepacia* complex	NC_015266.1	62.33% (YP_004306435.1)	80.58% (YP_004306434.1)
Burkholderia phages KS14	*B. cenocepacia*	NC_015273.1	52.53% (YP_004306862.1)	65.79% (YP_004306861.1)
Burkholderia phages ST79	*B. pseudomallei*	NC_021343.1	46.8% (YP_008060509.1)	0% (YP_008060510.1)
Burkholderia phages BEK	*B. pseudomallei*	CP008753.1	0%	99.05% (AIP84297.1)

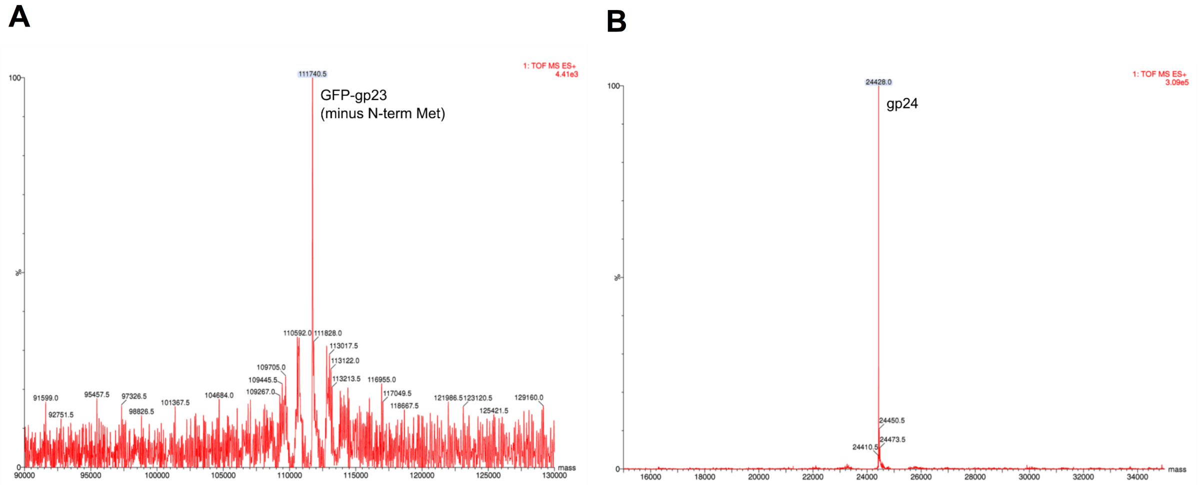

FIG S1 ESI-MS analysis of the 94TF bio-probe complex. Co-expressed GFP-tagged gp23 (A) and gp24 (B) were identified at their theoretical masses (111.8 kDa and 24.4 kDa, respectively) in solution after Ni-NTA purification.

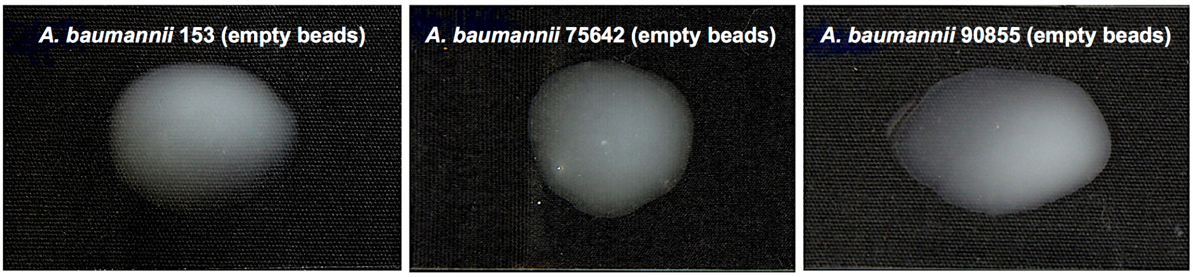

FIG S2 Lack of cross reactivity observed for *Acinetobacter baumannii* strains 153, 75642, 90855 when using 1% BSA-coated beads. These strains gave jelly-like positive agglutination during 94TF-LAA testing (as shown in Fig. 5A).

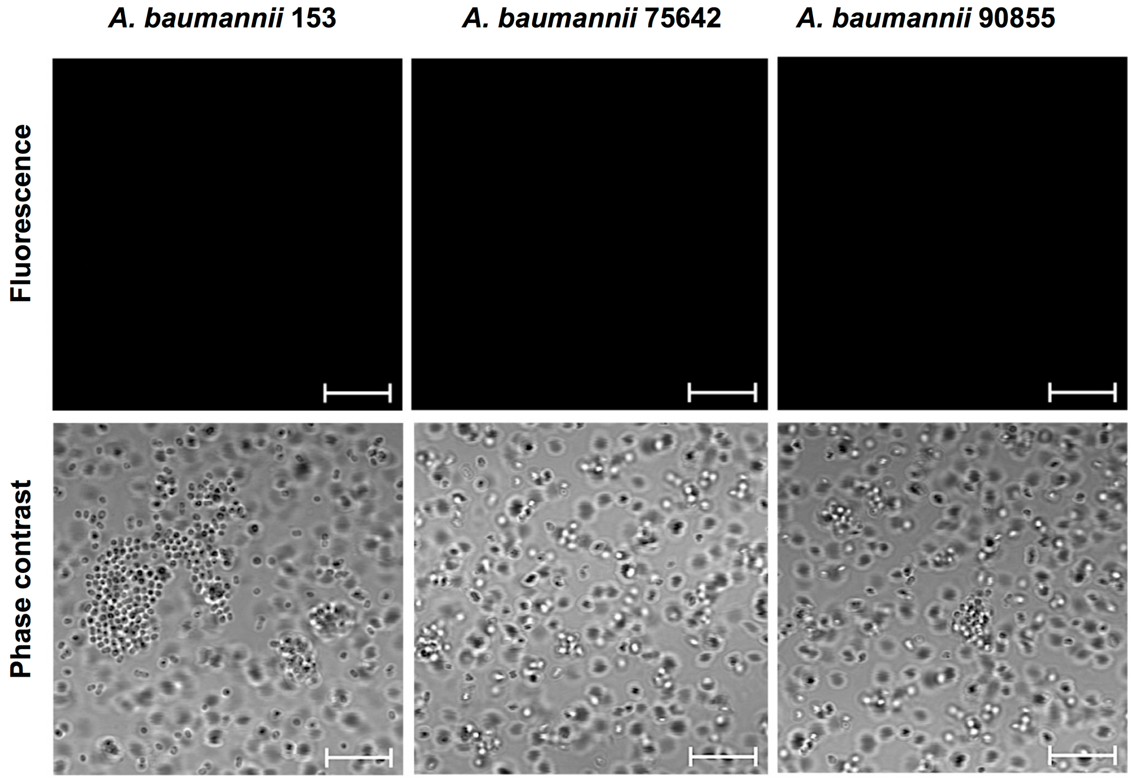

FIG S3 Confocal fluorescence micrographs showing lack of binding by GFP-94TF to *Acinetobacter baumannii* strains 153, 75642, and 90855 that gave false-positive results from the 94TF-LAA testing.

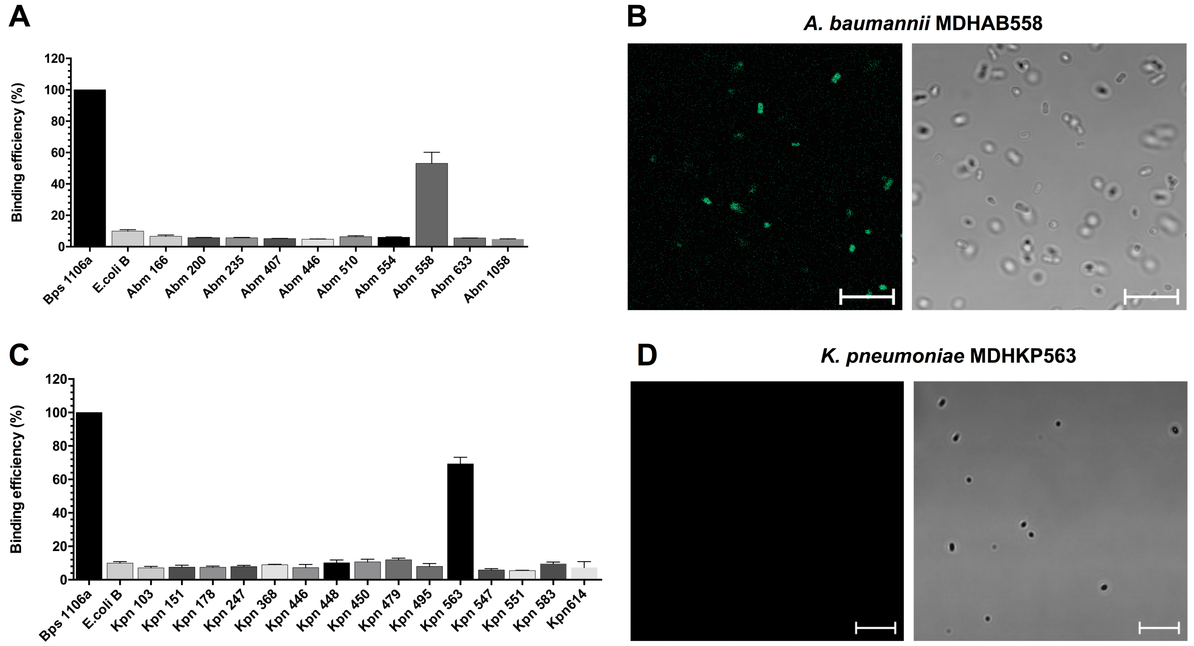

FIG S4 Cross-reactivity of GFP-94TF to *Acinetobacter baumannii* isolate MDHAB558 (A and B) and *Klebsiella pneumoniae* isolate MDHKP563 (C and D). In A and C, binding intensity was normalized against the binding observed for *B. pseudomallei* 1106a (set at 100%). Results represent the mean ± Standard Deviation (SD) of triplicate experiments. Scale bars in B and D indicate 10 µm.

Supplemental Material:

Biochemical-based detection of *B. pseudomallei.* Biochemical-based detection of *B. pseudomallei* was performed according to the ASM Sentinel Level Clinical Laboratory Guidelines for Suspected Agents of Bioterrorism and Emerging Infectious Disease and Clinical Microbiology Procedure Handbook and following ISO/IEC 17025 guidelines. In brief, clinical samples such as sputum, pus, urine and body fluids were directly cultured for bacteria using chocolate agar (CHOC) and MacConkey agar (MAC) and incubated at 37°C for a minimum of 48 hours. Blood cultures were grown using a BacT/Alert ™ automated blood culture system with positive-hemoculture bottles subsequently plated on blood agar, CHOC and MAC media. To ensure there was no influence on the identity of an isolate, all isolates were given an "unknown" tracking number. The 94TF-LAA and biochemical-based detection were performed in parallel from the same colony. Gram stains were performed on a thin-smear preparation in sterile 0.9% NaCl solution. Crystal violet, Lugol's iodine, acetone, and dilute carbol fuchsin were used. Gram reaction and bacterial shape were recorded. Oxidase test was performed by spreading a linear smear from the single colony on filter paper soaked with oxidase reagent. Biochemical reaction tubes including indole test, motility in semisolid medium, nitrate reduction test, oxidation and fermentation of glucose, maltose and lactose, citrate utilization, and the ability to reduce sulfur and ferment carbohydrates by triple sugar iron agar (TSI) were also tested. All biochemical reaction tubes were incubated in a temperature-controlled incubator at 37°C ± 1°C. Growth ability at 42°C was also performed. The biochemical reactions were read after 48 hours.

Antimicrobial susceptibility tests were performed against amikacin, colistin and amoxicillin/clavulanic acid (10-µg disc; Oxoid™, UK) with zones of inhibition measured according to recommendations from the Clinical and Laboratory Standards Institute (CLSI).

The field test was performed for eleven months (09/2019 to 07/2020) with the results of both assays collected and analyzed at the end of the study period.

Mass spectrometry (MS) analysis of protein species at FGCZ, Switzerland

LC-ESI-MS

Samples were 2-fold diluted with 1% TFA and transferred to autosampler vials for LC/MS. 10 µl of sample was injected into an ACQUITY UPLC@ BioResolve-RP-mAb 2.7µ 2.1x150 450 A (Waters, USA) column. For separation and elution on an Acquity UPLC station (Waters, USA), a gradient buffer A (0.1% FA in water)/ buffer B (0.1% FA in AN) at a flow rate 200ul/min at 500 C over 25 min was applied. The analysis was performed on a Synapt G2 mass spectrometer (Waters, UK) directly coupled with the UPLC station. Mass spectra were acquired in the positive-ion mode by scanning an m/z range from 100 to 4000 da with a scan duration of 1 s and an interscan delay of 0.1s. The spray voltage was set to 3 kV, the cone voltage to 50V, and source temperature 80 °C. The data were recorded with the MassLynx 4.2 Software (Waters, UK). For single peaks, the recorded m/z data were then deconvoluted into mass spectra by applying the maximum entropy algorithm MaxEnt1 (MaxLynx) with a resolution of the output mass 0.5 Da/channel and Uniform Gaussian Damage Model at the half height of 0.5 Da.

LC-MS-MS

Sample Preparation

Gel bands were cut in small pieces and washed twice with 100 µl of 100 mM ammonium bicarbonate:acetonitrile (50:50) and once with acetonitrile. The three supernatants were discarded. The proteins were digested using 10 µl of Sequencing Grade Trypsin (100 ng/µl in 10 mM HCl, Promega) and 30 µl of digestion buffer (10 mM Tris/2 mM CaCl2, pH 8.2). The

digestion was carried out in a microwave instrument (Discover System, CEM) for 30 min at 5 W and 60 °C. The supernatants were collected and the peptides were extracted from the gel pieces using 150 µl 0.1% trifluoroacetic acid / 50% acetonitrile (15 min in an ultrasonic bath). The supernatants were combined and the samples were finally dried in the speed-vac, resolubilized in 20 µl of 0.1% formic acid and centrifuged at max speed (20000 g) for 10 minutes. Ten microliters were transferred into LC-MS vials.

Liquid Chromatography-Mass Spectrometry Analysis

Mass spectrometry analysis was performed on a nanoAcquity UPLC (Waters Inc.) connected to a Q Exactive mass spectrometer (Thermo Scientific) equipped with a Digital PicoView source (New Objective). Solvent composition at the two channels was 0.1% formic acid for channel A and 0.1% formic acid, 99.9% acetonitrile for channel B. For each sample, 3 µl were injected. Peptides were trapped on a Symmetry C18 trap column (5 µm, 180 µm x 20 mm, Waters Inc.) and separated on a BEH300 C18 column (1.7 µm, 75 µm x 150 m, Waters Inc.) at a flow rate of 300 nL/min by a gradient from 5 to 35% B in 30 min, 60% B in 5 min and 80% B in 1 min. The mass spectrometer was operated in data-dependent mode (DDA), acquiring a full-scan MS spectra (350−1500 m/z) at a resolution of 70000 at 200 m/z after accumulation to a target value of 3000000, followed by HCD (higher-energy collision dissociation) fragmentation on the twelve most intense signals per cycle. HCD spectra were acquired at a resolution of 35000 using a normalized collision energy of 25 and a maximum injection time of 120 ms. The automatic gain control (AGC) was set to 50000 ions. Charge state screening was enabled and singly and unassigned charge states were rejected. Only precursors with intensity above 25000 were selected for MS/MS. Precursor masses previously selected for MS/MS measurement were excluded from further selection for 40 s, and the

exclusion window was set at 10 ppm. The samples were acquired using internal lock mass calibration on m/z 371.1010 and 445.1200.

Data Analysis

For the identification of interactors, the raw data were converted into Mascot Generic Format files (.mgf) using ProteoWizard (http://proteowizard.sourceforge.net/), and the proteins were identified using the Mascot search engine (Matrix Science, version 2.5.1.3). Spectra were searched against a Uniprot E. coli proteome database (taxonomy 83334, version from 2019-11-19), concatenated to its reversed decoyed fasta database. Methionine oxidation was set as variable modification, and enzyme specificity was set to trypsin allowing a maximum of two missed-cleavages. A fragment ion mass tolerance of 0.030 Da and a parent ion tolerance of 10.0 PPM were set. Scaffold (Proteome Software Inc., version 4.10) was used to validate MS/MS based peptide and protein identifications. Peptide identifications were accepted if they achieved a false discovery rate (FDR) of less than 0.1% by the Scaffold Local FDR algorithm. Protein identifications were accepted if they achieved an FDR of less than 1.0% and contained at least 2 identified peptides. The search engine PEAKS (PEAKS X, Bioinformatic Solutions) was used to confirm the presence of proteins GFP-gp23 and gp24 from their isolated gel bands. Data were searched with a fragment ion mass tolerance of 0.03 Da and a parent ion tolerance of 15.0 PPM. Oxidation (M) was specified as a variable modification. A maximum of 3 missed cleavages were allowed. Peptide identifications were accepted if they achieved a peptide false discovery rate (FDR) of less than 0.1% and the proteins contained at least 2 identified peptides.
